# Internal Guidelines for Reducing Lymph Node Contour Variability in Total Marrow and Lymph Node Irradiation

**DOI:** 10.3390/cancers15051536

**Published:** 2023-02-28

**Authors:** Damiano Dei, Nicola Lambri, Sara Stefanini, Veronica Vernier, Ricardo Coimbra Brioso, Leonardo Crespi, Elena Clerici, Luisa Bellu, Chiara De Philippis, Daniele Loiacono, Pierina Navarria, Giacomo Reggiori, Stefania Bramanti, Marcello Rodari, Stefano Tomatis, Arturo Chiti, Carmelo Carlo-Stella, Marta Scorsetti, Pietro Mancosu

**Affiliations:** 1 Department of Biomedical Sciences, Humanitas University, Via Rita Levi Montalcini 4, Pieve Emanuele, 20072 Milan, Italy; 2 Department of Radiotherapy and Radiosurgery, IRCCS Humanitas Research Hospital, via Manzoni 56, Rozzano, 20089 Milan, Italy; 3 Dipartimento di Elettronica, Informazione e Bioingegneria, Politecnico di Milano, 20133 Milan, Italy; 4 Health Data Science Centre, Human Technopole, 20157 Milan, Italy; 5 Department of Oncology and Hematology, IRCCS Humanitas Research Hospital, via Manzoni 56, Rozzano, 20089 Milan, Italy; 6 Department of Nuclear Medicine, IRCCS Humanitas Research Hospital, via Manzoni 56, Rozzano, 20089 Milan, Italy

**Keywords:** TMLI, guidelines, radiotherapy, interobserver variability, contour definition, clinical target volume, conditioning regimen, leukemia

## Abstract

**Simple Summary:**

The lymph node clinical target volume (CTV_LN) contour in total marrow and lymph node irradiation (TMLI) is extremely complex and time-consuming, involving the delineation of numerous lymph node chains. Furthermore, as few patients per year are usually treated, inter- and intraobserver variability pose a difficult challenge in CTV_LN definition. Currently, there is no globally accepted consensus on lymph node chain delineation for TMLI. In this study, we evaluated the impact of the introduction of internal guidelines for the lymph node contouring process in TMLI treatments. Both topological and dosimetric analyses were performed. Guidelines allowed for the reduction of variability in inter- and intra-CTV_LN delineation.

**Abstract:**

Background: The total marrow and lymph node irradiation (TMLI) target includes the bones, spleen, and lymph node chains, with the latter being the most challenging structures to contour. We evaluated the impact of introducing internal contour guidelines to reduce the inter- and intraobserver lymph node delineation variability in TMLI treatments. Methods: A total of 10 patients were randomly selected from our database of 104 TMLI patients so as to evaluate the guidelines’ efficacy. The lymph node clinical target volume (CTV_LN) was recontoured according to the guidelines (CTV_LN_GL_RO1) and compared to the historical guidelines (CTV_LN_Old). Both topological (i.e., Dice similarity coefficient (DSC)) and dosimetric (i.e., V95 (the volume receiving 95% of the prescription dose) metrics were calculated for all paired contours. Results: The mean DSCs were 0.82 ± 0.09, 0.97 ± 0.01, and 0.98 ± 0.02, respectively, for CTV_LN_Old vs. CTV_LN_GL_RO1, and between the inter- and intraobserver contours following the guidelines. Correspondingly, the mean CTV_LN-V95 dose differences were 4.8 ± 4.7%, 0.03 ± 0.5%, and 0.1 ± 0.1%. Conclusions: The guidelines reduced the CTV_LN contour variability. The high target coverage agreement revealed that historical CTV-to-planning-target-volume margins were safe, even if a relatively low DSC was observed.

## 1. Introduction

Total body irradiation (TBI) is a radiotherapy (RT) treatment that was developed in the late 1950s with the aim of increasing engraftment in bone marrow transplantation and facilitating the eradication of leukemic cells in acute myeloid leukemia, acute lymphoblastic leukemia, and in other hematologic malignancies [1]. The target of TBI is the whole body, thus exposing the patient to the risk of developing acute and late toxicity in the healthy tissues, especially in pediatric patients and in adult patients with comorbidities [2,3].

Over the years, the use of TBI has decreased due to (i) the long-term toxicities, (ii) the need for specific medical expertise, and (iii) the technical peculiarities of performing this complex treatment (a dedicated bunker, organ shielding devices, patient positioning, etc.). Furthermore, various therapeutic alternatives have emerged, such as chemotherapy-only myeloablative and reduced-intensity conditioning regimens, due to the discovery of the graft-versus-tumor effect in the context of allogeneic hematopoietic stem cell transplantation [4,5].

A recent phase III trial demonstrated the significant benefit of radiation in addition to chemotherapy in the pretransplant conditioning regimen, with a better overall survival of 0.91 compared to 0.75 at 2 years for patients who received chemo conditioning alone, as well as a lower incidence of relapse and treatment-related mortality [6].

The introduction of helical tomotherapy, intensity-modulated radiation therapy (IMRT), and later, volumetric modulated arc therapy (VMAT) techniques allowed the increasing of the dose conformity to the target while sparing the dose to the organs at risk (OARs) [7,8]. Intensity-modulated techniques were evaluated as replacements for standard TBI to irradiate hematopoietic tissue with a potential toxicity reduction to OARs [9,10,11,12,13,14,15,16]. Such approaches are referred to as total marrow and lymph node irradiation (TMLI) [17].

The TMLI target volume is defined as the patient’s bones, spleen, and lymph nodes. The definition of the clinical target volume (CTV) is crucial to performing an adequate treatment with intensity-modulated techniques. In particular, the delineation of the CTV of the lymph node chains (CTV_LN) is extremely complex and time-consuming due to its greater volume compared to standard RT treatments. Moreover, the definition of CTV_LN is more subject to observer variability since, typically, only a small number of patients with TMLI are treated each year in a few reference centers, resulting in a lack of multicenter studies.

Currently, there is no globally accepted consensus for the delineation of lymph node chains in TMLI, with studies in the literature usually referring to the inclusion of generic “major lymph node areas”. The clinical choice to exclude certain lymph node chains should take into account the expected toxicity for some sites, as already suggested for Waldeyer’s ring and the mesenteric lymph nodes [18,19].

In this study, we contoured the lymph node chains, starting from well-established international guidelines for specific regions, and we evaluated the impact of the introduction of internal contouring guidelines on the reduction of TMLI CTV_LN contour inter- and intravariability.

## 2. Materials and Methods

### 2.1. TMLI Procedure

Since 2010, in our center, 114 patients with pathologically proven hematological malignances, who had been identified as candidates for allogeneic transplantation, were treated with nonmyeloablative conditioning TMLI, with a prescribed dose of 2 Gy (1 fraction) [20].

All TMLI patients were immobilized in the supine position with arms along the body using an in-house, dedicated frame with multiple personalized masks [21,22]. For each patient, a free-breathing noncontrast computed tomography (CT) scan with a slice thickness of 5 mm was acquired using a BigBore CT system (Philips Healthcare, Best, Netherlands).

All TMLI plans were optimized for a VMAT technique and delivered on a TrueBeam LINAC (Varian Medical Systems, Palo Alto, CA, USA) [23,24]. The VMAT–TMLI plans were generated using either the progressive resolution optimization (PROIII v13) or the photon optimization (PO v15) algorithms (Varian Medical Systems), depending on the version available in the clinic at the time of the treatment. All dose calculations were performed using the Analytical Anisotropic Algorithm (AAA v.11–15).

The TMLI CTV included the bone marrow (CTV_BM), the spleen (CTV_Spleen), and all lymph node chains (CTV_LN). In our center, the CTV_BM was considered to be equal to the skeletal bones, adding the chest wall to the ribs to account for breathing motions. The mandible was excluded from the CTV_BM to reduce toxicity to the oral cavity, as were the hands since they have an extremely limited bone marrow presence. The total planning target volume (PTV_Tot) was defined as the Boolean operator “union” of three PTVs, obtained from the isotropic expansion of three corresponding CTVs, specifically: (i) PTV_BM = CTV_BM + 2 mm (+8 mm for arms and legs) to account for setup margin; (ii) PTV_Spleen = CTV_Spleen + 5 mm to account for breathing motions and setup margin; and (iii) PTV_LN = CTV_LN + 5 mm to account for target residual motion and setup margin.

Plans were normalized to PTV_Tot-D98% = 98% (i.e., 98% of PTV_Tot should receive 98% of the prescription dose). The lenses, eyes, brain, lungs, heart, kidneys, bowels, stomach, liver, rectum, and bladder were defined as OARs, and doses to these structures were minimized in the optimization process, following the ALARA (as low as reasonably achievable) principle. To adequately cover the PTV_Tot, a multi-isocenter approach was adopted for the plan optimization, using 5 isocenters for a total of 10 full arcs (360°). The collimator angle was set to 90°, with an asymmetric jaw aperture in the cranial–caudal direction, and a maximum aperture (~40 cm) in the left–right direction. Each arc overlapped with the adjacent ones for at least 2 cm to minimize the dose distribution uncertainty due to a potential patient misalignment. We refer to other studies for the full description of the protocol [22,25,26].

### 2.2. Target Definition Guidelines

For the first 104 TMLI patients, the CTV_LN was delineated by different radiation oncologists (ROs) using a nonwritten agreement.

In March 2022, a group consisting of the referent RO for the hematological diseases, two experienced ROs, an RO in training, and a hematologist, established written internal guidelines for the delineation of CTV_LN.

The present study collected several recommendations for each anatomical district. The criteria for CTV_LN delineation strictly followed international guidelines and lymph node CT atlas recommendations, including those from (i) Radiation Therapy Oncology Group (RTOG) [27]; (ii) International Association for the Study of Lung Cancer [28]; (iii) Offersen et al. [29]; (iv) Grégoire et al. [30]; and (v) Lengelé et al. [31]. In the target definition, a few lymph node chains, i.e., the most peripheral, were omitted from the target due to their lower involvement rate and in order to reduce potential toxicities. Due to the lack of consistent studies in the literature, this decision was based on our internal clinical experience and the consideration of a few other experiences as well [18,19]. The lymph node levels selected for each anatomical site are summarized in Table 1.

### 2.3. Guideline Evaluation

This study was divided into two parts. Initially, the agreement between the historical and the new CTV_LN contours was evaluated. Then, inter- and intraobserver variability was assessed for CTV_LN contoured following the guidelines.

A total of 10 patients were randomly selected (1 per year) from our internal database. A single expert radiation oncologist (RO1) recontoured the CTV_LN according to the guidelines (CTV_LN_GL_RO1). These new contours were compared to the historical lymph node target delineation (CTV_LN_Old).

The guidelines’ efficacy in reducing intra- and interobserver variability was prospectively assessed in, respectively, four and six patients. In detail, the CTV_LN_GL were re-contoured two times in blind mode, with a minimum interval of 2 months, by the same RO (CTV_LN_GL_RO1a and CTV_LN_GL_RO1b) to evaluate the guidelines’ intravariability, and by two independent ROs (CTV_LN_GL_RO1 and CTV_LN_GL_RO2) to evaluate the guidelines’ intervariability.

Each CTV_LN was split into four regions so as to investigate specific differences, including: H&N, thoracic, abdominal, and pelvic areas.

The sample was subdivided into three comparison groups (see Table 2): Group A (before vs. after guidelines’ introduction), Group B (interobserver variability), and Group C (intraobserver variability).

### 2.4. Data Analysis

The contours were compared by evaluating topological and dosimetric indexes.

#### 2.4.1. Topological Evaluation

The CVT_LN volumes, Dice similarity coefficient (DSC) values, mean distance-to-agreement (Mean DA), and Hausdorff distance (HD) were extracted for each case using an in-house script integrated into the RayStation Doctor (RaySearch Laboratories, Stockholm, Sweden) treatment planning system (TPS).

#### 2.4.2. Dosimetric Evaluation

For the retrospective and prospective patients, the VMAT plans were optimized using, respectively, the CTV_LN_Old and CTV_LN_GL_RO1 as part of the PTV_tot used for the optimization (see Section 2.1). Many dose-volume points were analyzed for each CTV_LN contour to assess the target coverage and guidelines’ consistency. In particular, the percentage of dose received by a specific percentage of volume (i.e., D90, D80) and the percentage of volume reached by a specific percentage of the prescription dose (i.e., V95, V90) were computed. Data were extracted using an in-house script developed for the Eclipse (Varian Medical System) TPS.

#### 2.4.3. Statistical Tests

Comparisons between the contour groups were performed using the Mann–Whitney test, while the Wilcoxon matched-pairs signed-rank test was used to compare dose-volume points between plans for the same patient. The threshold for statistical significance was set to *p* < 0.05. The analysis was performed using Python v 3.10.4 and the SciPy v 1.8.1 library.

## 3. Results

### 3.1. CTV_LN Inter-/Intraobserver Contouring Variability

**Figure 1:**
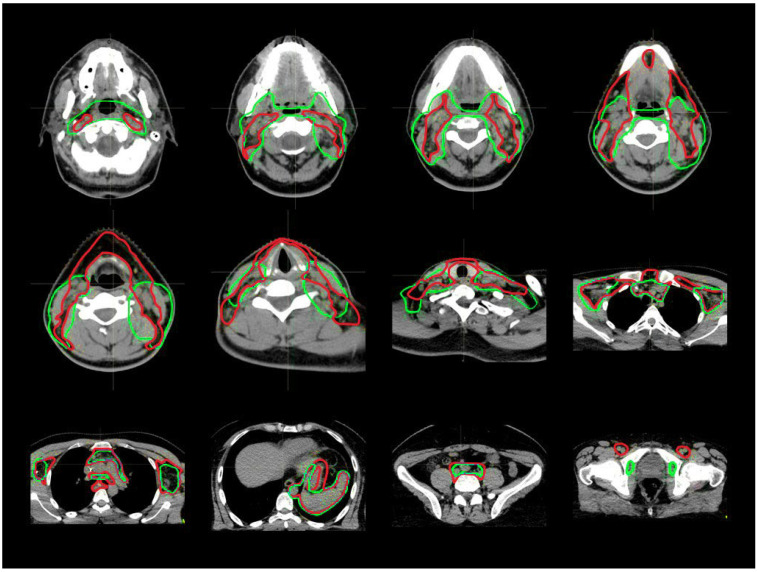
Axial view of CTV_LN contouring for every anatomical district, before (green segmentation) and after (red segmentation) the guidelines’ introduction.

**Figure 2:**
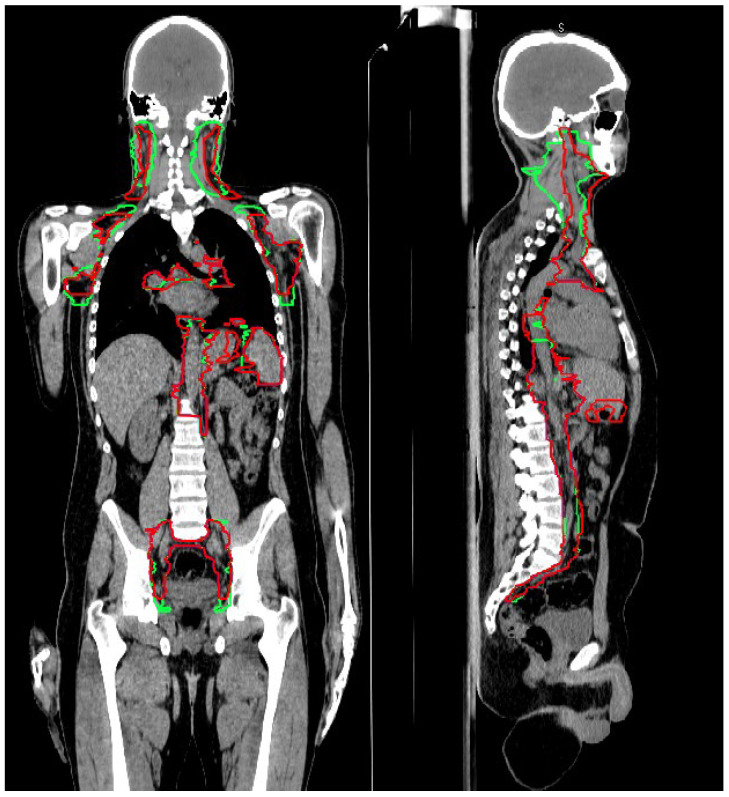
Coronal and sagittal views of CTV_LN contouring, before (green segmentation) and after (red segmentation) the guidelines’ introduction.

A total of 250 structures were analyzed. An example of target contouring before and after the introduction of the guidelines is shown as a representative case along with axial slices in Figure 1 and with coronal–sagittal views in Figure 2. In Figure S1 of the Supplementary Materials, a case with a partial chain miss in the H&N region is reported.

The mean CTV_LN volumes after the guidelines’ introduction increased from 2176 ± 600 cm^3^ to 2370 ± 672 cm^3^, due to the larger head and neck, gastric, and mediastinal lymph node delineation after the guidelines’ introduction (see Table 3).

The full topological analysis is reported in Table 4. For the retrospective patients, the CTV_LN mean DSC was 0.82 ± 0.09. The worst DSC result (0.69 ± 0.15) was observed for the H&N district. The thoracic lymph node contours showed a moderate variability, with a DSC of 0.77 ± 0.15, often caused by the sum of small differences in the delineation, especially for the hilum of the lung and between the mediastinal vessels. Finally, the abdominal and pelvic level delineations presented better reproducibility, with DSCs of, respectively, 0.82 ± 0.08 and 0.88 ± 0.09. Nonetheless, an uncertainty in delineating the gastric and bilateral inguinal lymph node regions was observed.

In the prospective inter- and intraobserver variability analyses, the mean DSCs were, respectively, 0.97 ± 0.01 and 0.98 ± 0.01, demonstrating an overall increase in contour agreement. The H&N region was the most affected by interobserver variability, leading to the lowest mean DSC, 0.88 ± 0.04. Mean DA and HD analyses confirmed the results of the DSC evaluation, with mean values of A being much larger than those of B or C. However, high standard deviations were observed for the mean DA and HD when compared to their respective mean values.

### 3.2. Target Coverage and Dose Distribution

The full dosimetric analysis is reported in Table 5. The V95 for CTV_LN_GL_RO1, CTV_LN_Old, CTV_LN_GL_RO2, and CTV_LN_GL_RO1b were, respectively, 94 ± 5%, 99 ± (<0.5)%, 99 ± 1%, 99 ± (<0.5)%, corresponding to CTV_LN-V95 dose differences of, respectively, 4.8 ± 4.7%, 0.3 ± 0.5%, and 0.1 ± 0.1%. Dose-coverage differences were significant in the Group A comparison, while no significant difference was observed within the inter- and intraobserver variability groups (i.e., Groups B and C).

Regarding the lymph node subregions, the V95 was >99% in all areas for Groups B and C, revealing the optimal dosimetric agreements after the introduction of the guidelines. On the contrary, the V95 values for Group A presented a large spread, ranging from 99% for the thorax and pelvis regions, to 91% and 89% for the H&N and Abdominal regions. For these two last cases, the V90 values increased by 2%.

## 4. Discussion

Phase I and II trials that included TMLI as part of the conditioning for bone marrow transplantation demonstrated encouraging engraftment, achieving full donor chimerism, a low incidence of graft-versus-host disease, and low extra-hematologic toxicities [10,17,20,25,32,33,34,35,36,37].

A controversial issue regarding TMLI organ sparing is a possible increase in extra-medullary relapses as compared to TBI. According to Kim et al., the only significant predictor of posttransplantation extra-medullary recurrence was the occurrence of this diffusion behavior before the transplantation [38]. Furthermore, the location of the relapse was not dose-dependent and could occur both in-field and out-field. Therefore, according to these findings, TMLI should not be linked to a higher incidence of extra-medullary relapse.

Several studies confirmed that RT is fundamental for hematological diseases, and they encouraged further improvements of TMLI treatment. This can be pursued both through technological developments and through the advancement of human knowledge. Particularly within the RT workflow, contouring remains a challenging and time-consuming task. To the best of our knowledge, this is the first study to investigate inter- and intraobserver variability in TMLI contouring.

TMLI is a highly modulated technique; therefore, target delineation is crucial. The variability in bone and spleen definition could be considered negligible, while CTV_LN delineation is subject to greater uncertainty. For this reason, in our hospital, we adopted a larger margin for the CTV_LN-to-PTV_LN expansion (5 mm), as compared to that of the bones (CTV_BM-to-PTV_BM, 2 mm). These margins were based on an internal analysis of the first patients included in the trial. For the spleen, the expansion of 5 mm in the three directions was performed to preserve lung function. The spleen is, in fact, subject to respiratory movements, which may require margins greater than 5 mm in the cranial–caudal direction. In our experience, the union of the PTVs (which, in addition to the spleen, includes the PTV_LN and the PTV_bones) allowed for complete coverage of the caudal region of the spleen in all of our treated patients, while for the cranial part, our clinical choice was to spare the left lung to limit pulmonary toxicity, accepting a possible limited uncovering of the spleen dome.

A potential approach to reducing operator-dependent uncertainty is to introduce an auto-segmentation tool, which has the advantages of harmonizing the contours and reducing manual segmentation variability. In a recent study regarding TMLI auto-segmentation techniques, a DSC of 0.73 ± 0.01 between manual and automatic segmentations for CTV_LN was reported [39], while in our analysis, the DSC, before and after the introduction of the guidelines, was higher (0.82 ± 0.09). In particular, the H&N district is confirmed to have high observer variability due to its anatomical complexity. Despite the worse agreement with manual contours, auto-segmentation approaches showed lower standard deviations (i.e., lower variability). However, an auto-segmentation tool needs robust contours for its training. To this aim, consensus guidelines could increase the consistency of the approach, potentially facilitating its applicability. Many studies showed that the introduction of guidelines helped to reduce inter- and intraobserver variability in CTV delineation in different anatomical regions [40,41,42]. In particular, the DAHANCA, EORTC, GORTEC, HKNPCSG, NCIC CTG, NCRI, NRG Oncology, and TROG consensus guidelines highlighted the clinical benefit of reducing interobserver variability through the implementation of training courses, the adoption of guidelines, and the proper use of the imaging examinations [30,40,43,44,45,46,47,48].

In the historical 3D-conformal planning approach, the target contouring variability had a minor impact on the dosimetric plan consistency due to the low intrinsic conformal dose to a concave target. Therefore, in this case, the introduction of target contouring guidelines would have benefits that were not as clear. New modulated delivery techniques, such as IMRT and VMAT, can irradiate a highly conformal dose distribution to the target volume while sparing neighboring healthy tissues. Lobefalo et al. showed that the introduction of guidelines in rectum cancer (i.e., a concave target) increased the mean PTV-V95 by 9.0% using a VMAT technique, while an increase of only 3.1% was observed for a classical “box” RT [49]. Therefore, modulated techniques applied to complex targets, such as TMLI, should benefit from an accurate and homogeneous definition of the target, reducing the delineation variability and improving the dose coverage.

Despite the absence of established international guidelines, the use of our written internal consensus allowed us to reduce CTV_LN inter- and intravariability, both in terms of topological and dosimetric indexes. The direct estimation of the CTV_LN contour consistency before the guidelines’ introduction was not possible. However, the greater DSC standard deviation for Group A (0.09) compared to Groups B (0.01) and C (0.02) is an indirect confirmation of the lower contour consistency. Furthermore, the DSC was significantly different for the A vs. C (*p* < 0.01) and A vs. B Groups (*p* = 0.03), as a possible consequence of low CTV_LN consistency in Group A. On the contrary, no significant difference was observed for Group B vs. Group C, showing low inter- and intravariability with the guidelines. The Group A vs. Group B comparison was significant only for the whole CTV_LN volume, while the nonsignificant values for the CTV_LN levels could be caused by the subjectivity in defining the cranial–caudal boundary and by the patients’ characteristics. For this reason, a comparison of CTV_LN districts between different patients with relatively different anatomies has a lower level of accuracy and is affected by greater uncertainty.

Areas with lower dose coverage were the most affected by the nonhomogeneity of lymph node delineation before the introduction of the guidelines. Specifically, the abdominal region resulted in a poor D90 = 92%, while the D80 = 102%. A possible explanation of the low D90 value could be the delineation of perigastric lymph nodes that were not systematically included before the guidelines’ introduction. Furthermore, the greater reproducibility in the delineation of the main lymph node chains is a possible consequence of the clear visualization on CT scans of the aorta, the iliac vessels, and their branches.

The dosimetric analysis showed that the retrospective treatment plans were clinically acceptable, despite the significant difference in dose coverage for Group A (the V95 dose difference in Group A was 4.8 ± 4.7%), and it revealed that the low CTV_LN delineation reproducibility (DSC = 0.82 ± 0.09) did not affect the overall plan quality. This is a possible consequence of the CTV-to-PTV expansion, as well as of the composition of the PTV_Tot (union of three overlapping PTVs), which decreases the impact of inter- and intravariability. Nonetheless, our data indicate that the use of guidelines can allow a reduction of the CTV_LN-to-PTV_LN margins for TMLI treatments.

Finally, the introduction of multi-imaging-based contouring is another possible approach to reducing inter- and intravariability [50,51]. This study was part of the AuToMI project, the aim of which is to spread the use of TMLI by improving clinical practices and introducing new, automated tools [23,24]. Further investigation will address the impact on workload due to the introduction of these internal guidelines and the application of automated segmentation tools. Moreover, a parallel study is currently underway to further decrease lymph node contouring uncertainty using co-registered, whole-body magnetic resonance imaging to enhance the CTV_LN individuation.

A few limitations should be taken into consideration when interpreting the results of this study. First, as this is a monocentric study, the generalizability of our findings to other institutions might be limited. Second, the definition of the lymph node chains was not based on an international consensus specific to TMLI, but on several international guidelines and our internal experience. Therefore, we do not recommend them as the definitive standard. However, our approach could be a useful starting point for specifying which major lymph node areas should be included in TMLI, and we suggest that international efforts should be made to standardize the definition of lymph node chains for this treatment. Finally, we did not use contrast media CT series, which may improve the definition of lymph node chains. Due to the frailty of transplant candidate patients and their susceptibility to chemo- and radio-related renal toxicity, we preferred to avoid the use of iodine-based agents.

## 5. Conclusions

This study revealed that the CTV_LN-to-PTV_LN historical margins were safe. The introduction of guidelines reduced the intra- and interobserver variability in CTV_LN delineation and dose coverage, which could potentially support lymph node margin reduction in future TMLI treatments, thus reducing the dose to healthy tissues.

## Figures and Tables

**Table 1:** Lymph nodes included in the CTV_LN, divided per anatomical site.

Region	Lymph Nodes
H&N	VIIa + VIIb + II + Ib + V + III + Ia + VIa + VIb + IVa + IVb
axilla	I + II + III + IV
mediastinum	1–8 + 10
abdomen	I + II + III
pelvis	I-VII + superficial and deep groin

**Table 2:** Scheme of the comparisons performed in this study.

CTV_LN Comparison	Comparison Abbreviation	Explanation
GL_RO1 vs. Old	A	After-GL vs. before-GL
GL_RO1 vs. GL_RO2	B	Inter-observer-variability
GL_RO1a vs. GL_RO1b	C	Intra-observer-variability

Legend: “GL_RO1”: contour performed by an expert RO after the introduction of the guidelines; “Old”: contour performed before the guidelines; “GL_RO2”: contour performed by a second expert RO after the introduction of the guidelines; “RO1a” and “RO1b”: two contours performed by the same expert RO after the introduction of the guidelines.

**Table 3:** CTV_LN and lymph node substructure volume analysis, before and after the guidelines’ introduction; the minimum and maximum values are reported in parentheses.

	Before GL [cm^3^]	After GL [cm^3^]
CTV_LN_Tot	2176 ± 600 [1312–3194]	2370 ± 672 [1412–3511]
CTV_LN_H&N	332 ± 128 [197–558]	559 ± 111 [180–544]
CTV_LN_Thorax	501 ± 178 [254–801]	532 ± 200 [265–853]
CTV_LN_Abdominal	710 ± 196 [454–1057]	721 ± 260 [582–1407]
CTV_LN_Pelvis	612 ± 175 [350–898]	590 ± 177 [349–871]

Legend: CTV_LN: lymph node clinical target volume; GL: Guidelines.

**Table 4:** Topological analysis of CTV_LN for each comparison group: (A) before vs. after guidelines’ introduction; (B) interobserver variability; (C) intraobserver variability.

	DSC			*p*-Value (DSC)			Mean DA [mm]			HD [mm]		
LN levels	A	B	C	A vs. B	A vs. C	B vs. C	A	B	C	A	B	C
**Tot**	0.82 ± 0.09	0.97 ± 0.01	0.98 ± 0.02	**0.03**	**<0.01**	1.00	0.4 ± 0.2	0.1 ± 0.1	0.03 ± 0.02	7 ± 1	2 ± 2	1.9 ± 0.3
H&N	0.69 ± 0.15	0.88 ± 0.04	0.96 ± 0.03	0.27	**<0.01**	0.13	0.5 ± 0.4	0.1 ± 0.1	0.02 ± 0.01	7 ± 7	2 ± 1	0.9 ± 0.3
Thorax	0.77 ± 0.15	0.97 ± 0.01	0.97 ± 0.02	0.18	**0.02**	1.00	0.5 ± 0.5	0.1 ± 0.2	0.03 ± 0.01	6 ± 7	2 ± 2	1.4 ± 0.5
Abdomen	0.82 ± 0.08	0.98 ± 0.01	0.97 ± 0.01	0.05	**0.02**	0.35	0.7 ± 0.4	0.1 ± 0.2	0.03 ± 0.01	8 ± 6	1 ± 2	1.6 ± 0.6
Pelvis	0.88 ± 0.09	0.96 ± 0.01	0.95 ± 0.03	0.27	0.16	0.80	0.2 ± 0.2	0.1 ± 0.2	0.06 ± 0.04	3 ± 2	1 ± 1	1.6 ± 0.5

Legend: CTV_LN: lymph node clinical target volume; DA: distance-to-agreement; DSC: Dice similarity coefficient; HD: Hausdorff distance. Significant *p*-value results of Mann–Whitney test for DSC comparisons between groups are highlighted in bold.

**Table 5:** Dosimetric parameter analysis for each comparison group: (A) before vs. after guidelines, (B) interobserver variability, (C) intraobserver variability. The values reported are normalized to the prescribed dose.

	**D90**						**D80**					
CTV_LN	A		B		C		A		B		C	
	RO1	Old	RO1	RO2	RO1a	RO1b	RO1	Old	RO1	RO2	RO1a	RO1b
Tot	**1.01 ± 0.11**	**1.03 ± 0.06**	1.04 ± 0.02	1.04 ± 0.02	1.04 ± 0.01	1.04 ± 0.01	**1.04 ± 0.06**	**1.05 ± 0.05**	1.06 ± 0.02	1.060.02	1.06 ± 0.01	1.06 ± 0.01
H&N	**0.98 ± 0.09**	**1.03 ± 0.05**	1.05 ± 0.02	1.05 ± 0.02	1.04 ± 0.01	1.04 ± 0.01	**1.03 ± 0.06**	**1.05 ± 0.05**	1.08 ± 0.02	1.08 ± 0.02	1.06 ± 0.02	1.06 ± 0.02
Thorax	**1.03 ± 0.09**	**1.04 ± 0.07**	1.05 ± 0.02	1.05 ± 0.02	1.04 ± 0.02	1.04 ± 0.02	**1.05 ± 0.07**	**1.05 ± 0.07**	1.07 ± 0.02	1.07 ± 0.02	1.06 ± 0.02	1.06 ± 0.02
Abdomen	**0.92 ± 0.18**	**1.03 ± 0.07**	1.03 ± 0.02	1.03 ± 0.01	1.04 ± 0.01	1.04 ± 0.01	**1.02 ± 0.11**	**1.05 ± 0.07**	1.05 ± 0.02	1.06 ± 0.01	1.05 ± 0.01	1.05 ± 0.01
Pelvis	**1.04 ± 0.06**	**1.04 ± 0.06**	1.06 ± 0.02	1.06 ± 0.02	1.06 ± 0.02	1.05 ± 0.02	**1.05 ± 0.06**	**1.06 ± 0.06**	1.07 ± 0.02	1.07 ± 0.02	1.07 ± 0.02	1.07 ± 0.02
	**V95**						**V90**					
	A		B		C		A		B		C	
CTV_LN	RO1	Old	RO1	RO2	RO1	RO1b	RO1	Old	RO1	RO2	RO1	RO1b
Tot	**0.94 ± 0.05**	**0.99 ± (<<)**	0.99 ± (<<)	0.99 ± 0.01	1.00 ± (<<)	0.99 ± (<<)	**0.96 ± 0.04**	**1.00 ± (<<)**	0.99 ± (<<)	0.99 ± (<<)	1.00 ± (<<)	1.00 ± (<<)
H&N	**0.91 ± 0.05**	**0.99 ± 0.01**	0.99 ± 0.01	0.99 ± 0.01	0.99 ± (<<)	0.99 ± (<<)	**0.93 ± 0.04**	**1.00 ± (<<)**	0.99 ± (<<)	1.00 ± 0.01	1.00 ± (<<)	1.00 ± (<<)
Thorax	**0.99 ± 0.05**	**1.00 ± (<<)**	1.00 ± (<<)	1.00 ± (<<)	1.00 ± (<<)	0.99 ± (<<)	**0.99 ± 0.03**	**1.00 ± (<<)**	1.00 ± (<<)	1.00 ± (<<)	1.00 ± (<<)	1.00 ± (<<)
Abdomen	**0.89 ± 0.09**	**1.00 ± (<<)**	1.00 ± 0.01	1.00 ± 0.01	1.00 ± (<<)	1.00 ± (<<)	**0.91 ± 0.08**	**1.00 ± (<<)**	1.00 ± (<<)	1.00 ± (<<)	1.00 ± (<<)	1.00 ± (<<)
Pelvis	**0.99 ± 0.03**	**1.00 ± (<<)**	1.00 ± (<<)	1.00 ± 0.01	1.00 ± (<<)	0.99 ± (<<)	**0.99 ± 0.03**	**1.00 ± (<<)**	1.00 ± (<<)	1.00 ± (<<)	1.00 ± (<<)	1.00 ± (<<)

Legend: “± (<<)”: standard deviation <0.005; “GL_RO1”: contour performed by an expert RO after the guidelines’ introduction; “Old”: contour performed before the guidelines; “GL_RO2”: contour performed by a second expert RO after the guidelines’ introduction; “RO1a” and “RO1b”: two contours performed by the same expert RO. In bold are highlighted values for which the Wilcoxon signed-rank test between groups had a *p*-value < 0.05.

## Data Availability

The data presented in this study are available on request from the corresponding author.

## Supplementary Materials

The following supporting information can be downloaded at: https://www.mdpi.com/article/10.3390/cancers15051536/s1, Figure S1: Partial chain miss.
