# Supplementary material for: Internal Guidelines for Reducing Lymph Node Contour Variability in Total Marrow and Lymph Node Irradiation
Source: Cancers (Basel). 2023 Feb 28;15(5):1536. doi: 10.3390/cancers15051536 (PMC10000500; doi:10.3390/cancers15051536)
Supplement: Supplementary file 1 [file cancers-15-01536-s001.zip › cancers-2129813-supplementary.pdf]

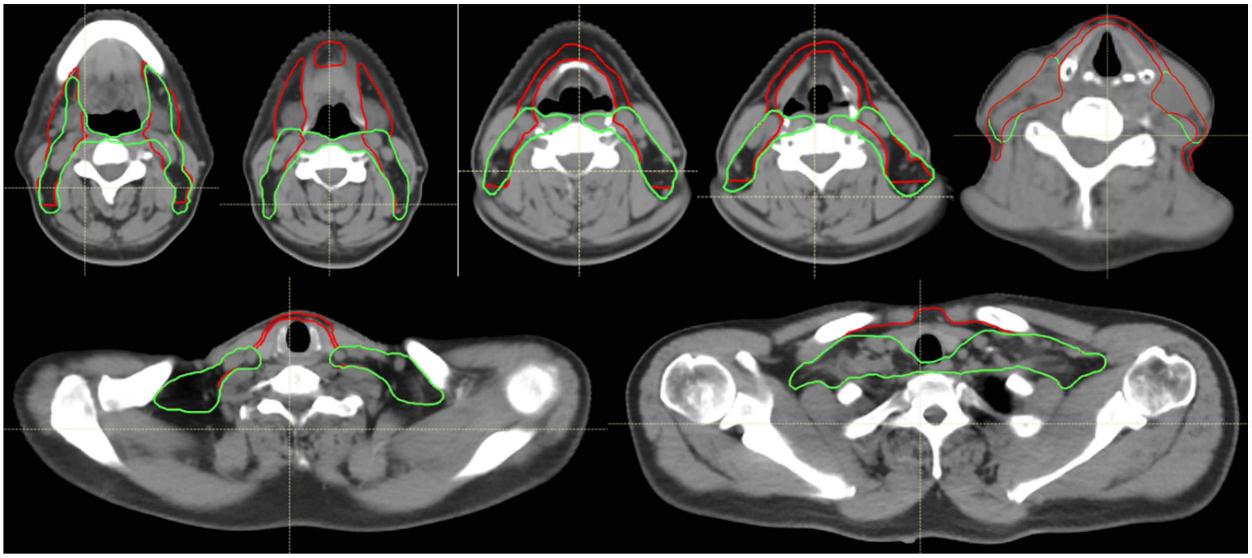

Figure S1: Partial chain miss. Representative case with a partial chain miss in the H&N region before (green segmentation) and after (red segmentation) the guidelines' introduction.
